# Biased instantaneous regional muscle activation maps: Embedded fuzzy topology and image feature analysis

**DOI:** 10.3389/fbioe.2022.934041

**Published:** 2022-12-22

**Authors:** Carlos De la Fuente, Alejandro Weinstein, Alejandro Neira, Oscar Valencia, Carlos Cruz-Montecinos, Rony Silvestre, Patricio A. Pincheira, Felipe Palma, Felipe P. Carpes

**Affiliations:** ^1^ Carrera de Kinesiología, Departamento de Cs. de la Salud, Facultad de Medicina, Pontificia Universidad Católica, Santiago, Chile; ^2^ Laboratory of Neuromechanics, Universidade Federal do Pampa, Campus Uruguaiana, Uruguaiana, Brazil; ^3^ Unidad de Biomecánica, Centro de Innovación, Clínica MEDS, Santiago, Chile; ^4^ Centro de Investigación y Desarrollo en Ingeniería en Salud, Universidad de Valparaíso, Valparaíso, Chile; ^5^ Escuela de Kinesiología, Facultad de Ciencias, Universidad Mayor, Santiago, Chile; ^6^ Laboratorio Integrativo de Biomecánica y Fisiología del Esfuerzo, Facultad de Medicina, Escuela de Kinesiología, Universidad de los Andes, Santiago, Chile; ^7^ Laboratory of Clinical Biomechanics, Department of Physical Therapy, Faculty of Medicine, Universidad de Chile, Santiago, Chile; ^8^ School of Health and Rehabilitation Science, The University of Queensland, Brisbane, QLD, Australia; ^9^ School of Human Movement and Nutrition Sciences, The University of Queensland, Brisbane, QLD, Australia

**Keywords:** muscle, high-density electromyography, UMAP, entropy, barycenter, image moments, segmentation

## Abstract

The instantaneous spatial representation of electrical propagation produced by muscle contraction may introduce bias in surface electromyographical (sEMG) activation maps. Here, we described the effect of instantaneous spatial representation (sEMG segmentation) on embedded fuzzy topological polyhedrons and image features extracted from sEMG activation maps. We analyzed 73,008 topographic sEMG activation maps from seven healthy participants (age 21.4 ± 1.5 years and body mass 74.5 ± 8.5 kg) who performed submaximal isometric plantar flexions with 64 surface electrodes placed over the medial gastrocnemius muscle. Window lengths of 50, 100, 150, 250, 500, and 1,000 ms and overlap of 0, 25, 50, 75, and 90% to change sEMG map generation were tested in a factorial design (grid search). The Shannon entropy and volume of global embedded tri-dimensional geometries (polyhedron projections), and the Shannon entropy, location of the center (LoC), and image moments of maps were analyzed. The polyhedron volume increased when the overlap was <25% and >75%. Entropy decreased when the overlap was <25% and >75% and when the window length was <100 ms and >500 ms. The LoC in the x-axis, entropy, and the histogram moments of maps showed effects for overlap (*p* < 0.001), while the LoC in the y-axis and entropy showed effects for both overlap and window length (*p* < 0.001). In conclusion, the instantaneous sEMG maps are first affected by outer parameters of the overlap, followed by the length of the window. Thus, choosing the window length and overlap parameters can introduce bias in sEMG activation maps, resulting in distorted regional muscle activation.

## Introduction

Physical therapists, biomechanists, and engineers regularly infer (quantitative or qualitative interpretation) neuromuscular adaptations from surface electromyography (sEMG) activation maps (Vieira et al., 2011; Campanini et al., 2020; Merletti and Cerone, 2020). An sEMG activation map represents the discrete distribution of the voltage propagation elicited from the train sum of motor unit action potentials (MUAPs) collected from an array of electrodes on the skin (Botter and Vieira, 2015; Guzmán-Venegas et al., 2015; Ghaderi and Marateb, 2017; Jordanić et al., 2017; Vigotsky et al., 2017; Merletti and Muceli, 2019; Merletti and Cerone, 2020). Thus, multiple electrodes allow for obtaining sEMG activation maps that can be interpreted as images (Jordanić et al., 2017; Merletti and Muceli, 2019), similar to brain activation (Beniczky and Schomer, 2020) or a uterus electromyogram (Xu et al., 2022). Each map pixel corresponds to the voltage acquired by each electrode. Thus, the map can be defined by $I_{i,j,t}=\sqrt{\frac{1}{N-1}\;\overset{N}{\underset{k=1}{\sum }}{\left({EMG\left[nT\right]}_{i,j}\;w\left[n-mR\right]\right)}_{k}^{2}}$ . $I_{i,j,t}$ is the pixel intensity that represents the magnitude of the muscle activity located at (i,j), t is the number of maps obtained after windowing, ${EMG\left[nT\right]}_{i,j}$ is the sEMG signal located in the array, $w\left[n\right]$ is the window or epoch, N is the length of $w\left[n\right]$ , and R is the hop size that determines the amount of overlap.

Traditionally, sEMG activation map quantification involves feature extraction, where the location of the center (LoC or barycenter) and the Shannon entropy are the most used (Guzmán-Venegas et al., 2015). The LoC is defined by $LoC=\frac{\sum_{i,j}I_{i,j\;}\left[\begin{array}{c} i\\ j \end{array}\right]}{\sum_{i,j}I_{i,j\;}}$ (Jordanic et al., 2016; Pincheira et al., 2020). Meanwhile, the entropy that explores homogeneity is defined by $E=-\sum_{k=1}^{N}{p\left(k\right)}^{2}\log_{2}{p\left(k\right)}^{2}$ . ${p\left(k\right)}^{2}$ is the probability of the square of the root mean square value at electrode k (Farina et al., 2008). In addition, image moments (expected value, variance, skewness, and kurtosis) can also describe image changes in the spatial time domain (Brown and Godman, 2011).

On the other hand, several conditions might introduce undesired dispersion and noise. Therefore, capturing latent map data might be convenient for understanding how synthetic distortions are introduced. The latent data, which retain lower-dimension information that explains higher-dimension data, have been optimized through the Uniform Manifold Approximation and Projection (UMAP) algorithm (McInnes et al., 2020; Ali et al., 2019; Oskolkov, 2019). UMAP projects a fuzzy topological set of high dimensions equivalent to low-dimensional data (McInnes et al., 2020; Ali et al., 2019). The approximation is possible by creating fuzzy topological projections with binary cross-entropy and projections (McInnes et al., 2020). The binary cross-entropy is modeled by $\sum_{je\in E}\left[w_{h}\left(e\right)\;\log\frac{W_{h}\left(e\right)}{W_{l}\left(e\right)}+\left(1-w_{h}\left(e\right)\right)\;\log\left(\frac{1-W_{h}\left(e\right)}{1-W_{l}\left(e\right)}\right)\;\right]$ , while the weight between neighbors is modeled by w=e−dxi−xj−ρiσ . $\rho_{i}$ is the distance from the *i*-th data points to its first nearest neighbor (Oskolkov, 2019). The first term ensures fuzzy connectivity (simplex or node connections). In contrast, the second term does not permit the creation of simplexes (McInnes et al., 2020). Hence, UMAP might allow the topological representation of different sEMG maps (high dimensions) resulting from N and R parameters.

Previously, sEMG segmentation influenced the electrical manifestation of fatigue conclusions (De la Fuente et al., 2021). Since sEMG activation maps depend inherently on segmentation, alterations are expected in the sEMG activation map. However, there is still large variability in choosing window lengths, i.e., 50 ms, 100 ms, 150 ms, 250 ms, 500 ms, or 1,000 ms (Botter and Vieira, 2015; Guzmán-Venegas et al., 2015; Jordanic et al., 2016; Falla et al., 2017; Jordanić et al., 2017; Zhu et al., 2017; Martinez-Valdes et al., 2018; Vinti et al., 2018; Watanabe et al., 2018; Hegyi et al., 2019), and truncation methods (non-overlapping (Guzmán-Venegas et al., 2015; Falla et al., 2017; Jordanić et al., 2017)). Therefore, understanding how segmentation may distort regional muscle activation is still a concern. Here, we aimed to describe the effect of instantaneous spatial representation (sEMG segmentation) on embedded fuzzy topological polyhedrons and image features extracted from the sEMG activation maps obtained with high-density sEMG on healthy participants performing a submaximal isometric contraction of medial gastrocnemius.

## Materials and methods

### Study design

**FIGURE 1:**
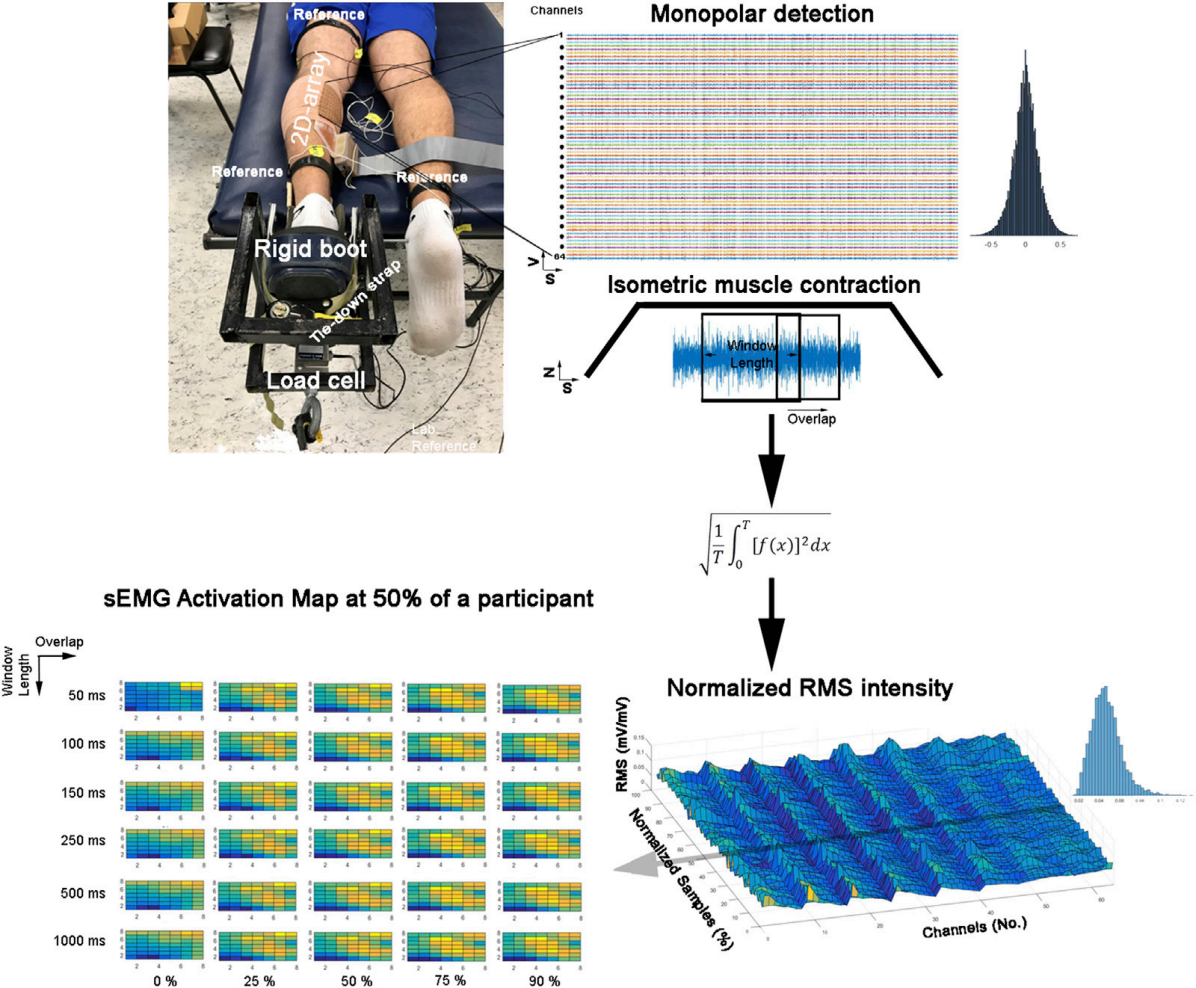
Experimental set-up and instantaneous surface electromyographic map generation flow. Colors represent the instantaneous potential amplitude distribution (yellow indicates more intensity, and blue indicates lower intensity).

We conducted a factorial experiment to test 30 signal processing conditions (Figure 1). The sample included 73,008 sEMG activation maps obtained from seven healthy participants (aged 21.4 ± 1.5 years, body mass 74.5 ± 8.5 kg, height 1.77 ± 0.01 m, and body mass index 20.9 ± 2.2 kg/m^2^) who performed a submaximal isometric plantar flexion with the ankle at neutral position (60% with the ankle in a 90° position) in a controlled laboratory set-up (Figure 1). Here, we considered the medial gastrocnemius muscle as a good muscle model due to its application in clinics and biomechanics and because it was previously used in EMG segmentation (Theisen et al., 2016; De la Fuente et al., 2021). The Bioethics Committee of the Andes University (Santiago, Chile) approved this study (# INV-IN201701), which was developed according to the principles of the Declaration of Helsinki. All participants signed a consent term agreeing to participate in this study.

### Data

A total of 73,008 sEMG activation maps were included in the study (data are available in https://www.researchgate.net/publication/365904692_Biased_instantaneous_regional_muscle_activation_maps_embedded_fuzzy_topology_and_image_features_analysis_datapart1 and https://www.researchgate.net/publication/365904985_Biased_instantaneous_regional_muscle_activation_maps_embedded_fuzzy_topology_and_image_features_analysis_datapart2). They were the result of 39 experiments of a total of 42 experiments (6 trials x 7 participants). Of the 42 experiments, three experiments were excluded due to artifacts. These 39 experiments contained 10,240 samples x 64 channels. The 73,008 sEMG activation maps resulted in combining 1,872 maps and 39 experiments. The 1,872 maps resulted from 30 conditions, that is, window lengths (50, 100, 150, 250, 500, and 1,000 ms) combined with an overlap (0%, 25%, 50%, 75%, and 90%) without repetition. This combination resulted in 100, 136, 199, 423, and 1,014 maps (Pincheira et al., 2021).

### Experimental set-up

Participants were lying prone on a bench with their hip, knee, and ankle in a neutral joint position. The ankle of the participants was tightly strapped to a customized rigid structure (Figure 1). Then, they were asked to perform three maximal voluntary isometric contractions against a force transducer placed at a metatarsal head level (Revere Transducers^®^, 9363-B10-500-20T1R, United States). Each attempt lasted 5 seconds, with a 3-min rest period between repetitions (a period of non-contraction to recover basal muscle energy conditions). Immediately, the participants were asked to perform the submaximal voluntary contraction. The contractions were sustained for 20 s, and the duration of the ascending/descending ramps was 6 s and 8 s, respectively, for the hold phase. The participants received real-time visual feedback, displaying a trapezoid target (Figure 1). The participants completed six trials.

### Electrode location and data acquisition

Prior to data acquisition, the skin was shaved, abrased (Everi: Spes Medica s. r.l, Battipaglia, Italy), and cleaned with alcohol to diminish the skin impedance. Then, a semi‐disposable adhesive with 64 electrodes organized in eight rows and eight columns of 1 mm diameter and an inter-electrode distance of 10 mm was attached (GR10MM0808, OT Bioelettronica, Torino, Italy) (Pincheira et al., 2021). The electrode spaces were filled with a conductive cream (Spes Medica s.r.l., Italy) (Pincheira et al., 2021).

The electrode was placed over the most prominent region of the medial gastrocnemius, and the muscle belly was determined by palpation during a resisted plantar flexion. Then, the electrode was aligned in the cephalocaudal axis with respect to the line formed between the medial femoral condyle and malleolus. For the mediolateral axis, the electrode was aligned with respect to the medial contour of the medial gastrocnemius muscle. The superomedial electrode corner was fixed at 30% of the distance of the cephalocaudal axis, as was described previously (Pincheira et al., 2021).

A total of 64 monopolar sEMG signals were collected from the electrodes, amplified with a gain of 200, and digitized at a sampling frequency of 2,048 Hz with a 12-bit resolution and 3-dB bandwidth 10–500 Hz (EMG-USB2: OTBioelettronica, Turin, Italy). The reference electrodes were positioned according to Pincheira et al. (2021) over the contralateral ankle and superior to the electrode near the popliteal fossa (Pincheira et al., 2021). Two additional reference electrodes were placed on the tibial tuberosity and the fibula to improve the EMG signal-to-noise ratio. Once the quality of the signals was assured, the electrodes were firmly secured with an elastic adhesive bandage (Figure 1).

Correct electrode placement was confirmed by assessing sEMG signals online for low baseline noise levels and possible artifacts, cortocircuit, or bad contact during visual inspection during brief plantar flexion contractions (Pincheira et al., 2021). The signal was evaluated at rest (without contraction) and under contraction (Pincheira et al., 2021). Non-saturated signals and out-of-power line interference were appreciated during the acquisition (Pincheira et al., 2021). Nevertheless, three experiments were excluded during offline signal processing after observing in the time and frequency domains. The domains showed increased noise.

### Pre-processing

The sEMG signals were mean-centered to zero and segmented at the force plateau signal. Then, the signals were filtered by a zero-lag second-order Butterworth with a bandpass of 20–400 Hz. Outlier channels were manually identified and confirmed using the Z-score. A mean with 1 pixel of radio was assigned for outlier pixels from channels with confirmed higher Z-scores (<0.01% was assigned). Afterward, the sEMG signals were convolved with a rectangular window. Our convolved sEMG signals were arranged in a matrix 8 × 8, and the maps were normalized to the maximum value of the whole matrixes during the plateau (Figure 1).

### Window length and overlap (intervention)

The window lengths were chosen based on previous reports (Guzmán-Venegas et al., 2015; Jordanic et al., 2016; Falla et al., 2017; Jordanić et al., 2017; Zhu et al., 2017; Martinez-Valdes et al., 2018; Watanabe et al., 2018; Hegyi et al., 2019). The overlap parameters were 0, 25, 50, 75, and 90%, resulting in 30 different combinations between the window length and overlap $\left\{\left(50,0\right),\left(50,25\right),\ldots ,\left(1000,75\right),\left(1000,90\right)\right\}$ to introduce variability to the sEMG activation maps to study its effects.

### Uniform manifold approximation and projection and feature image extraction

The sEMG activation maps of each condition of all participants were concatenated [73,008 × 64] and introduced to the UMAP algorithm version 1.4.1 (Meehan et al., 2020). The global structure of high-dimensional data (64 dimensions) was embedded into three-dimensional data (McInnes et al., 2020; McInnes, 2018). The number of neighbors was 10, the minimum distance was 0.7, the number of components was three dimensions, the metric was Euclidean, the number of epochs was 200, the learning rate was 1, local connectivity was 1, repulsion strength was 1, the spread was 1, the fuzzy set operation was 1, and the negative sample rate was 5. After assessing the level of connectivity and homogeneity of the structures, we created a 3D polyhedron (finite elements) to obtain their volume and Shannon entropy. In addition, we extracted the image features from sEMG activation maps, LoC, Shannon entropy, and image moment (expected value–moment 1-, variance–moment 2-, skewness–moment 3-, and kurtosis–moment 4-) (Brown and Godman, 2011).

### Variables

The following continuous variables were determined: 1) volume of the fuzzy topological structure obtained from the embedded dataset and normalized to a maximum value, 2) entropy of the fuzzy topological structure from the embedded dataset obtained as the Shannon entropy (Farina et al., 2008), 3) LoC obtained from the sEMG activation map in both x and y coordinates (Jordanić et al., 2017), 4) Shannon entropy obtained from the sEMG activation maps (Farina et al., 2008), 5) moment-1 of maps obtained from the sEMG activation map as the expected value (Brown and Godman, 2011), 6) moment-2 of maps obtained from the sEMG activation map as variance (Brown and Godman, 2011), 7) moment-3 of maps obtained from the sEMG activation map as skewness (Brown and Godman, 2011), and 8) moment-4 of maps obtained from the sEMG activation map as kurtosis (Brown and Godman, 2011).

### Data analysis

The sEMG activation maps were described as the expected value and variance. Normality and homoscedasticity assumptions were checked prior to the image analysis feature using two-way ANOVA 2 × 5 × 6 (two factors: window length and overlap; six levels of the length of windows: 50 ms, 100 ms, 150 ms, 250 ms, 500 ms, and 1,000 ms; and five levels of sliding: 0%, 25%, 50%, 75%, and 90%) for main effects. Effect sizes were described as the square sum of the effects divided by the total sum of squares to show the explained variance [small: η^2^ < 0.04 (< 4%), medium: between 0.04 (4%) and 0.64 (64%), and large: > 0.64 (64%) (Ferguson, 2009)]. The Tukey–Kramer test was used to find differences between groups. The K-medoid algorithm was applied to explore the differences between factors. The number of clusters with K-medoids was evaluated as the sum of the ratio between the sum of that within the Euclidean distance and Euclidean distance of each point with their medoid found. Then, the elbow method before convergence was chosen. The alpha error was equal to 0.05 for all statistics. The volume and entropy behavior were studied using a non-linear least square method, and fuzzy sEMG polyhedrons were described in the UMAP space. The zero-crossing of the fitted curve was described. All calculi were made through MATLAB software (MathWorks, Inc., United States).

## Results

**FIGURE 2:**
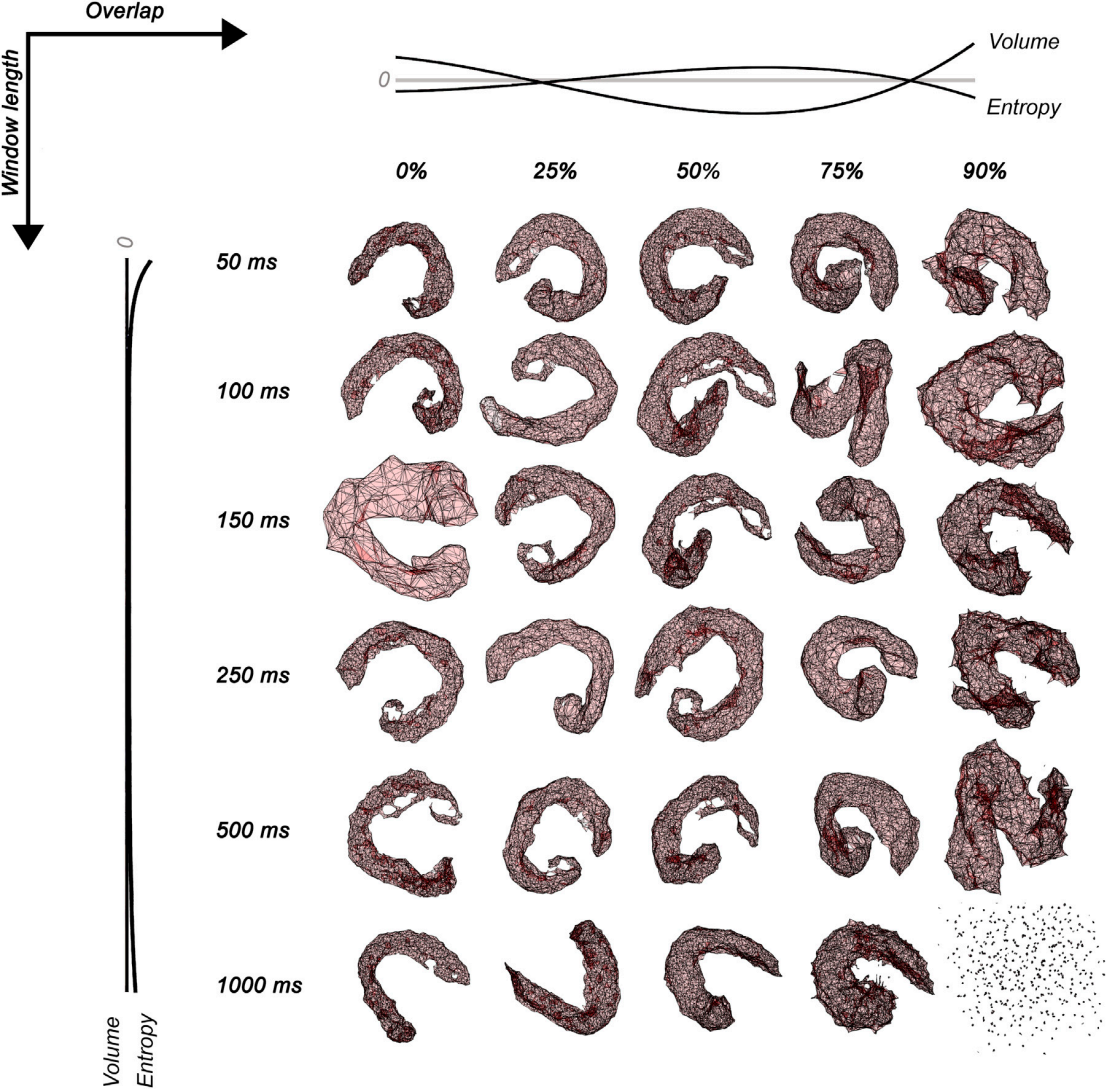
Surface electromyographic maps embedded in three dimensions for 30 conditions of signal processing varying window lengths (50 ms, 100 ms, 150 ms, 250 ms, 500 ms, and 1,000 ms) and overlap (0, 25, 50, 75, and 90%). The normalized no-linear fitting for the Shannon entropy and volume of sEMG polyhedrons is also shown in dark lines. Notice that the combination of 1000 ms and 90% of overlap projects no simplexes connections.

The polyhedron volume increased when the overlap was <25% and >75%. Entropy decreased when the overlap was <25% and >75% and when the window length was <100 ms and >500 ms. The polyhedron volume R^2^ was 73.5% and 16.9% for overlap and window length, respectively. The polyhedron entropy R^2^ was 90.1% and <1% for overlap and window length, respectively. The polyhedron zero-crossing for volume in the overlap was at 25%, and between 75% and 90%. The polyhedron zero-crossing for entropy in the overlap was between 25% and 50%, and between 75% and 90%. Non-zero crossings were found for window lengths. Figure 2 shows the volume and entropy behavior of embedded sEMG activation maps.

Map LoC_x_ (Table 1) showed a main effect for both overlap (*p* < 0.001, η^2^ = 0.998, large effect size) and window length (η2 < 0.04, small effect size), and there was interaction (*p* < 0.001, η^2^ < 0.04, small effect size). Overlap showed differences between all multiple comparisons (*p* < 0.001). Window length showed multicomparison differences between 50 ms and all window lengths (*p* < 0.001). Data were grouped into five clusters with centroids: 3.2 mm, 7.7 mm, 16.4 mm, 24.1 mm, and 32.8 mm. Map LoC_y_ (Table 1) showed only a main effect for overlap (*p* < 0.001, η^2^ = 0.998, large effect size). There were multiple comparison differences between all overlaps (*p* < 0.001), and data were grouped into six clusters with centroids: 3.5 mm, 8.2 mm, 17.6 mm, 25.9 mm, 34.5 mm, and 35.9 mm.

**TABLE 1:** Topographical EMG map outcomes.

	50 ms	100 ms	150 ms	250 ms	500 ms	1,000 ms
LoCx, m.m.						
0%	33 (0.013)	33 (0.013)	33 (0.010)	33 (0.010)	33 (0.010)	33 (0.010)
25%	24 (0.007)	24 (0.006)	24 (0.005)	24 (0.005)	24 (0.005)	24 (0.005)
50%	16 (0.003)	16 (0.003)	16 (0.002)	16 (0.002)	16 (0.002)	16 (0.002)
75%	08 (7.4e-4)	08 (5.7e-4)	08 (5.4e-4)	08 (5.2e-4)	08 (5.2e-4)	08 (5.1e-4)
90%	03 (1.2e-4)	03 (9.7e-5)	03 (9.2e-5)	03 (8.9e-5)	03 (8.7e-5)	03 (8.7e-5)
LoCy, m.m.						
0%	35 (0.012)	35 (0.010)	35 (0.010)	35 (0.010)	35 (0.009)	35 (0.009)
25%	26 (0.007)	26 (0.005)	26 (0.005)	26 (0.005)	26 (0.005)	26 (0.005)
50%	18 (0.003)	18 (0.003)	18 (0.002)	18 (0.002)	18 (0.002)	18 (0.002)
75%	08 (6.6e-4)	08 (5.5e-4)	08 (5.2e-4)	08 (5.0e-4)	08 (5.0e-4)	08 (4.9e-4)
90%	03 (1.2e-4)	03 (9.4e-5)	03 (8.9e-5)	03 (8.6e-5)	03 (8.5e-5)	03 (8.4e-5)
Entropy, d.u.						
0%	3.11 (0.012)	3.11 (0.012)	3.12 (0.012)	3.11 (0.012)	3.11 (0.012)	3.11 (0.023)
25%	3.11 (0.012)	3.11 (0.012)	3.11 (0.012)	3.11 (0.012)	3.11 (0.012)	3.11 (0.012)
50%	3.11 (0.012)	3.11 (0.012)	3.11 (0.012)	3.11 (0.012)	3.11 (0.012)	3.11 (0.012)
75%	3.11 (0.009)	3.11 (0.009)	3.11 (0.009)	3.11 (0.009)	3.11 (0.009)	3.11 (0.009)
90%	2.97 (7.1e-4)	2.97 (7.1e-4)	2.97 (5.1e-4)	2.97 (4.8e-4)	2.98 (4.6e-4)	2.98 (4.7e-4)
Moment-1, d.u.						
0%	0.126 (0.004)	0.128 (0.004)	0.128 (0.004)	0.128 (0.004)	0.128 (0.004)	0.129 (0.004)
25%	0.126 (0.004)	0.128 (0.004)	0.128 (0.004)	0.128 (0.004)	0.128 (0.004)	0.129 (0.004)
50%	0.126 (0.004)	0.128 (0.004)	0.128 (0.004)	0.128 (0.004)	0.128 (0.004)	0.129 (0.004)
75%	0.126 (0.004)	0.128 (0.004)	0.128 (0.004)	0.128 (0.004)	0.128 (0.004)	0.129 (0.004)
90%	0.126 (0.004)	0.128 (0.004)	0.128 (0.004)	0.128 (0.004)	0.128 (0.004)	0.129 (0.004)
Moment-2 x 10^−5^, d.u.						
0%	2.14 (8.84e-11)	1.04 (3.71e-11)	1.03 (3.45e-11)	1.03 (3.25e-11)	1.03 (3.20e-11)	1.03 (3.19e-11)
25%	1.89 (8.00e-11)	1.33 (4.77e-11)	1.33 (4.60e-11)	1.33 (4.43e-11)	1.33 (4.39e-11)	1.33 (4.38e-11)
50%	2.14 (8.84e-11)	1.28 (4.33e-11)	1.28 (3.91e-11)	1.28 (3.78e-11)	1.28 (3.73e-11)	1.28 (3.72e-11)
75%	2.24 (7.84e-11)	1.33 (3.99e-11)	1.33 (3.58e-11)	1.33 (3.44e-11)	1.33 (3.40e-11)	1.33 (3.39e-11)
90%	2.33 (7.95e-11)	1.33 (3.99e-11)	1.33 (3.57e-11)	1.33 (3.43e-11)	1.33 (3.39e-11)	1.33 (3.38e-11)
Moment-3, d.u.						
0%	−0.01 (0.41)	0.03 (0.42)	0.04 (0.41)	0.05 (0.41)	0.05 (0.41)	0.05 (0.41)
25%	−0.01 (0.41)	0.03 (0.43)	0.05 (0.43)	0.05 (0.43)	0.06 (0.42)	0.06 (0.42)
50%	0.00 (0.42)	0.03 (0.42)	0.05 (0.41)	0.05 (0.42)	0.06 (0.41)	0.06 (0.41)
75%	0.00 (0.43)	0.03 (0.42)	0.05 (0.42)	0.05 (0.42)	0.06 (0.41)	0.06 (0.41)
90%	0.00 (0.43)	0.03 (0.42)	0.06 (0.42)	0.06 (0.41)	0.06 (0.41)	0.06 (0.41)
Moment-4, d.u.						
0%	2.47 (0.80)	2.52 (0.82)	2.52 (0.83)	2.53 (0.83)	2.54 (0.85)	2.55 (0.85)
25%	2.46 (0.82)	2.49 (0.79)	2.50 (0.82)	2.52 (0.83)	2.52 (0.83)	2.52 (0.83)
50%	2.48 (0.80)	2.51 (0.80)	2.52 (0.82)	2.53 (0.82)	2.54 (0.83)	2.54 (0.83)
75%	2.47 (0.79)	2.50 (0.79)	2.51 (0.81)	2.52 (0.81)	2.53 (0.82)	2.53 (0.81)
90%	2.47 (0.79)	2.50 (0.80)	2.51 (0.81)	2.52 (0.81)	2.53 (0.82)	2.53 (0.82)

d.u. = dimensionless unit.

Data are expressed as the expected value (E[x]) of the histogram and variance of the expected value (E[x - E[x]]^2^).

Map entropy (Table 1) showed a main effect for both overlap (*p* < 0.001, η^2^ = 0.998, large effect size) and window length (*p* < 0.001, η^2^ < 0.04, small effect size), and there was interaction (*p* < 0.001, η^2^ < 0.04, small effect size). There were multiple comparison differences between 50 ms and all window lengths (*p* < 0.001). Data were grouped into five clusters with centroids: 1.8 d.u., 2.4 d.u., 2.9 d.u., 3.0 d.u., and 4.3 d.u.

Map moment-1 (Table 1) showed a main effect for the window length (*p* < 0.001, η^2^ < 0.04, small effect size). There were differences between 50 ms and 1,000 ms (*p* = 0.036), 50 ms and 150 ms (*p* = 0.004), 50 ms and 250 ms (*p* = 0.001), 50 ms and 500 ms (*p* = 0.001), and 50 ms and 1000 ms (*p* < 0.004). Data were grouped into five clusters with centroids: 0.06 d.u., 0.09 d.u., 0.13 d.u., 0.18 d.u., and 0.24 d.u. Map moment-2 (Table 1) showed a main effect for the window length (*p* < 0.001, η^2^ < 0.04, small effect size). There were differences between 50 ms and all window lengths (*p* < 0.001). Data were grouped into one cluster. Map moment-3 (Table 1) showed a main effect for the window length (*p* < 0.001, η^2^ < 0.04, small effect size). There were differences between 50 ms and all window lengths (*p* < 0.001), 100 ms and 150 ms (*p* = 0.005), and 100 ms and the rest of the window lengths (*p* < 0.001). Data were grouped into one cluster. Map moment-4 (Table 1) showed a main effect for the window length (*p* < 0.001, η^2^ < 0.04, small effect size). There were differences between 50 ms and the rest of the window lengths (*p* < 0.001), 100 ms and 250 ms (*p* = 0.006), 100 ms and 500 ms (*p* < 0.001), 100 ms and 1,000 ms (*p* < 0.001), and 150 ms and 1000 ms (*p* = 0.017). Data were grouped into one cluster.

## Discussion

The most important finding in our study was that the sEMG segmentation parameters (overlap and window length) of activation maps introduce bias, resulting in distorted regional muscle activation compromising the map inferences. For example, we can conclude about regional sEMG activation with or without clear regional sEMG activation when there were not, e.g., the statistical error types (Akobeng, 2016). The topological dimensional reduction and feature extraction of the sEMG maps confirmed it. Outer segmentation parameters tested here have caused the highest distortion in the activation maps; independently, no-overlap and small window length trends reduce the activation map region, while large overlap and window length trends increase the activation map region. Thus, sEMG map generation can modify the spatial myoelectrical activity and should be carefully considered by their physiological and clinical repercussions, i.e., wrong rehabilitation or performance planning. Furthermore, many clinical and sport science studies did not fully consider it in the past, and there is high variability in the choice of segmentation parameters (Botter and Vieira, 2015; Guzmán-Venegas et al., 2015; Jordanic et al., 2016; Falla et al., 2017; Jordanić et al., 2017; Zhu et al., 2017; Martinez-Valdes et al., 2018; Vinti et al., 2018; Watanabe et al., 2018; Hegyi et al., 2019) and truncation use (Stadler et al., 2007; Guzmán-Venegas et al., 2015; Falla et al., 2017; Jordanić et al., 2017).

The high-dimensional sEMG maps embedded into a low-dimensional dataset were studied through their entropy and volume. These variables permitted an understanding of three regions of activation. Overlap showed an increased volume and decreased entropy at outer parameters (two regions) and increased entropy with low volume at central parameters (one region). The window length showed decreased entropy at outer parameters (two regions) and higher entropy at central parameters (one region), while the volume trended to be constant. The entropy of sEMG polyhedrons quantified the geometrical heterogeneity of the embedding data (Franch et al., 2019), which represents the chance to order the fuzzy nodes projected from the RMS of MUAPs spatially distributed in our study. Thus, the decreased entropy shows a most regular geometry (homogeneity) due to decreased local connectivity (McInnes et al., 2020; Sánchez-Rico and Alvarado, 2019), which occurred with a large volume, suggesting more distance between nodes (less similar RMS of MUAPs). Consequently, there was less chance to order the fuzzy nodes projected from the RMS of MUAPs. This last distorted muscle activation suggests that two scenarios occurred in the outer parameters, an attenuated map for small overlap and window length, where there was a more significant proportion of low RMS of MUAPs (blue pixels; please visualize the sEMG maps of Figure 1), and a blurred map for large overlap and window length, where there was a more significant proportion of high RMS of MUAPs (yellow pixels; please visualize the sEMG maps of Figure 1).

On the other hand, an increased entropy shows a most irregular geometry (heterogeneity) due to increased local connectivity (McInnes et al., 2020; McInnes, 2018; Sánchez-Rico and Alvarado, 2019), which occurred with a small volume suggesting a lower distance between nodes (more similar RMS of MUAPs). Central parameters with higher entropy and lower volume were found near 50% overlap, while for window length, higher entropy and lower volume were found between 100 ms and 500 ms. A case of the total loss of connectivity was found for 1,000 ms, and 90% of overlap in coherence with findings of gene studies using UMAP (please, see Figure 2) (Dorrity et al., 2020).

Regarding the extracted features from sEMG activation maps, the LoC_x_, LoC_y_, and entropy confirmed a main distorted effect of the overlap on maps. The clustering analysis permitted decomposing data in coherence with the multiple comparison results. For y-coordinates, six clusters were found, suggesting that overlap 0% had two centroids, meaning that there were two sub-groups of 50 ms. For x-coordinates and entropy, five clusters were found in coherence with overlapping. Regarding the window length, only the x-coordinate and entropy showed differences (small effect size). In consequence, the 50 ms without overlap generated the most dissimilar sEMG map. These findings agree with discontinuities that can be introduced by small window lengths and the artifacts caused without window sliding (Yip et al., 2017). This last issue is caused by truncation ringing (Gibbs artifact), where small windowing abruptly magnifies intensity changes like a high-pass filter (Stadler et al., 2007). Thus, overlapping and small windowing can be an essential source to create a synthetic bias on the sEMG activity distorting the MUAP visualization techniques (Stadler et al., 2007; Vigotsky et al., 2017).

Finally, the image moments changed the sEMG activation maps but with a small effect size. This change suggests a lower sensitivity of image moments to detect biased sEMG maps compared to UMAP, LoC, and entropy of map features. The main limitation to the current study was the sEMG available grid used, which is related to the level of the spatial resolution of the sEMG intensity maps. The space aliasing was set according to our available electrode (inter-electrode distance of 10 mm). The standard acquisition of sEMG map indicates a relative acceptable use of 10 mm and sampling frequency in space higher than 200 samples/m (Merletti and Muceli, 2019; Merletti and Cerone, 2020). Also, the maximal spatial sampling may be appreciated using 90% of the spatial power density distribution on the x-axis, y-axis, or both (Afsharipour et al., 2019). However, electrodes lower or equal to 8 mm would obtain better spatial resolution. Although there are many options for selecting the shape of the window function, we used a rectangular one as a fixed and controlled experimental factor. Here, the effect of the window type on myoelectric manifestations is outside the scope of our study, and these limitations have been addressed in a previous publication (Tan and Jiang, 1984). The pinnate architecture of medial gastrocnemius limits our results only for this kind of muscle.

## Conclusion

Here, we demonstrate that embedded sEMG maps and features of image extraction change the spatial muscle activation by segmentation parameters. The instantaneous sEMG maps were primarily affected by outer parameters of the overlap, followed by the outer parameters of the window length. Consequently, choosing the window length and overlap parameters can introduce bias in sEMG activation maps, resulting in distorted regional muscle activation.

## Data Availability

Original datasets are available in a publicly accessible repository: (1) https://www.researchgate.net/publication/365904692_Biased_instantaneous_regional_muscle_activation_maps_embedded_fuzzy_topology_and_image_features_analysis_datapart1 and (2) https://www.researchgate.net/publication/365904985_Biased_instantaneous_regional_muscle_activation_maps_embedded_fuzzy_topology_and_image_features_analysis_datapart2.
